# Common variations in *ALG9 *are not associated with bipolar I disorder: a family-based study

**DOI:** 10.1186/1744-9081-2-25

**Published:** 2006-07-21

**Authors:** Bora E Baysal, Joan E Willett-Brozick, Silviu-Alin Bacanu, Sevilla Detera-Wadleigh, Vishwajit L Nimgaonkar

**Affiliations:** 1 Department of Obstetrics, Gynecology and Reproductive Sciences, The University of Pittsburgh School of Medicine, University of Pittsburgh, Pittsburgh, PA 15213, USA; 2 Department of Human Genetics, Graduate School of Public Health, University of Pittsburgh, Pittsburgh, PA 15261, USA; 3 Department of Psychiatry, The University of Pittsburgh School of Medicine, University of Pittsburgh, Pittsburgh, PA 15213, USA; 4 GlaxoSmithKline, Moore Drive, Research Triangle Park, NC 27709, USA; 5 National Institute of Mental Health, National Institutes of Health, Bethesda, MD 20892, USA

## Abstract

**Background:**

A mannosyltransferase gene (*ALG9*, *DIBD1*) at chromosome band 11q23 was previously identified to be disrupted by a balanced chromosomal translocation t(9;11)(p24;q23) co-segregating with bipolar affective disorder in a small family. Inborn ALG9 deficiency (congenital disorders of glycosylation type IL) is associated with progressive microcephaly, seizures, developmental delay, and hepatomegaly. It is unknown whether common variations of *ALG9 *predispose to bipolar affective disorder.

**Methods:**

We tested five polymorphic markers spanning *ALG9 *(three intragenic and one upstream microsatellite repeats and one common missense variation, V289I (rs10502151) for their association with bipolar I disorder in two pedigree series. The NIMH (National Institute of Mental Health) pedigrees had a total of 166 families showing transmissions to 250 affected offspring, whereas The PITT (The University of Pittsburgh) pedigrees had a total of 129 families showing transmissions to 135 cases. We used transmission disequilibrium test for the association analyses.

**Results:**

We identified three common and distinct haplotypes spanning the *ALG9 *gene. We found no statistically-significant evidence of transmission disequilibrium of marker alleles or multi-marker haplotypes to the affected offspring with bipolar I disorder.

**Conclusion:**

These results suggest that common variations in *ALG9 *do not play a major role in predisposition to bipolar affective disorder.

## Background

**Figure 1:**
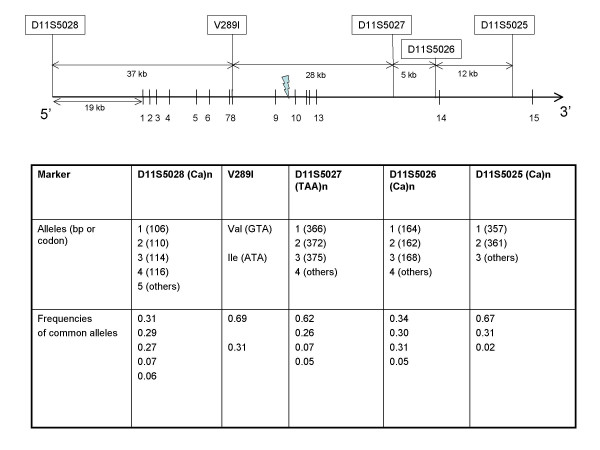
**A schematic view of the *ALG9 *gene structure and the relative locations of the five polymorphic markers**. Vertical lines represent the exons and lightening bolt represents the location of the translocation breakpoint. Relative positions of the exons and the markers are approximately in scale. The bottom part contains a table that describes common alleles of the markers, which were used in the transmission disequilibrium test, and their frequencies in the PITT sample.

Family, twin and adoption studies indicate a strong genetic contribution to the etiology of bipolar affective disorder (BPAD) [1]. The genetics of bipolar disorder, however, is complex and probably involves multiple genes interacting with each other and environmental components. Currently, susceptibility genes for BPAD and their mechanism of action in disease predisposition are largely unknown. Identification of certain susceptibility genes for BPAD could be facilitated by studying rare families where the disease co-segregates with a chromosomal abnormality among multiple affected relatives [2]. Previously, we described a family with five individuals affected with Bipolar I disorder in three generations and one individual with recurrent unipolar depression [3]. All six affected individuals were carriers of a balanced chromosomal translocation t(9;11)(p24;q23). Although five other individuals carrying the translocation were clinically normal at the time of the diagnostic interview, the absence of any affected individuals without the translocation suggested reduced penetrance of the chromosomal aberration in the causation of BPAD in this family. Subsequently, we captured and characterized the breakpoint junctions of both derivative chromosomes 9 and 11 using somatic cell hybridization methods [4]. We found that a mannosyltransferase gene, *ALG9 (*Asparagine-Linked Glycosylation mutant 9;*DIBD1, D*isrupted *I*n *B*ipolar *D*isorder *1*), at chromosome 11q23 was physically disrupted by the translocation breakpoint [5]. The translocation disrupted *ALG9*, which is composed of 15 exons spread to approximately 82 kb genomic distance, between exons 9 and 10 (Figure 1). No gene was disrupted at the chromosome band 9p24 breakpoint.

*ALG9 *encodes a mannosyltransferase protein which is involved in the synthesis of lipid-linked oligosaccharides (LLOs) in the protein N-glycosylation pathway [6]. *ALG9 *is ubiquitously expressed in different anatomical regions of the central nervous system. N-linked glycosylation is essential for the modification of proteins in eukaryotes and is one of the most complex metabolic pathways known, involving more than 40 biochemical steps. Congenital disorders of glycosylation type I (CDG-I), an increasingly recognized and expanding group of genetic conditions, are caused by abnormalities in the synthesis of the LLOs or in their attachment to proteins [7]. Most affected children have multi-system involvement indicating the importance of normal protein glycosylation in cellular function. CDG are clinically associated with a broad range of symptoms including the failure to thrive, hypotonia, cerebellar dysfunction, seizures, mental retardation, and coagulopathy.

The deletion of the *ALG9 *gene in yeast results in accumulation of LLOs and in hypoglycosylation of secreted proteins, suggesting that the *ALG9 *protein product functions in the addition of terminal α-1, 2- or α-1, 6-mannoses in the endoplasmic reticulum during the stepwise assembly of LLO [8]. Recently, the first human cases of *ALG9 *deficiency (congenital disorders of glycosylation type IL; CDG-IL), caused by E523K [9] and Y286C [10] homozygous missense mutations, were described in two infants. The mutations caused an accumulation of lipid-linked-GlcNAc(2)Man(6) and -GlcNAc(2)Man(8) structures and led to transfer of incomplete oligosaccharides precursors to protein. Both infants exhibited progressive microcephaly, seizures, developmental delay, and hepatomegaly. Also, there were additional clinical features common to other forms of CDG, including cystic renal disease, pericardial effusion and tamponade, partial villous atrophy, hypoproteinemia, esotropia, inverted nipples, coagulopathy, and brain imaging findings that suggested cerebral and cerebellar atrophy. Systemic features of the affected cases were consistent with the ubiquitous gene expression pattern and the central nervous system defects suggested a critical role for *ALG9 *in neurogenesis or in neuronal survival. To test whether common variations in the *ALG9 *gene are associated with BPAD, we performed a family-based association analysis using two pedigree series.

## Methods

We previously evaluated linkage and linkage disequilibrium in the NIMH (National Institute of Mental Health) bipolar pedigrees [5]. The NIMH pedigrees include series 1 which consisted of 22 multiplex families that were previously used in chromosomal and genomic scans and series 2, which is a collection of 97 pedigrees from the NIMH Genetics Initiative for bipolar disorder [11]. In our earlier analyses, two hierarchical phenotype models, Affection Status Model (ASM) I and ASM II were used. ASM I included bipolar I, bipolar II with major depression, and schizoaffective illness, and ASM II included phenotypes in ASM I plus recurrent unipolar disorder. These analyses did not show evidence of statistically significant linkage or linkage disequilibrium to the *ALG9 *gene using ASM I and ASM II as diagnostic categories. Here, we extend our analyses of the *ALG9 *gene with a family-based study of genetic association in a new pedigree series that is composed of 133 small nuclear families that have been collected at the University of Pittsburgh (PITT). We confine our analyses to cases with a diagnosis of bipolar I disorder, because this was the diagnosis assigned to the five translocation carriers in the original translocation pedigree. The PITT series contain exclusively bipolar I cases. We also include the NIMH series after recoding of the diagnoses: bipolar I cases are recoded as "affected" and all other psychiatric diagnoses as "unknown" (Table 1). The collection and genetic study of the PITT families were approved by the University of Pittsburgh IRB committee.

**Table 1:** Characteristics of the bipolar pedigree series analyzed for association with *ALG9*

Pedigree Series	Structure of pedigrees	Total No. of nuclear families	Total No. of subjects in the series	No. of families with transmissions to affected offspring	Total No. of bipolar I cases
PITT	trios, duos, sib pairs with two, one or no parents	133	424	129	135
NIMH	Multi-generational with multiple affected subjects	306	1903	166	250

Genotyping was performed using four simple tandem repeat polymorphisms (STRPs) and one missense single nucleotide polymorphisms [5]. Our earlier sequence analysis of 60 subjects with BPAD provided us with a polymorphism profile of the gene. A non-synonymous single nucleotide polymorphism (SNP), V289I (rs10502151), had the highest minor allele frequency (0.35) among the four SNPs discovered. The SNP, V289I (rs10502151), in exon 8 of *ALG9 *was genotyped by PCR amplification and restriction enzyme digestion as described earlier [5]. The STRPs pDJ159O1-GT2 (D11S5025; GenBank: AF113397), -GT1 (D11S5026; GenBank: AF113398), -TAT (D11S5027; GenBank: AF113399), and -CA (D11S5028; GenBank: AF113400) were originally developed during fine mapping of the hereditary paraganglioma type 1 critical region at chromosome band 11q23 [12] and later mapped in and around the *ALG9 *gene. The information on the allelic distributions of the STRPs is available in Genome Database. These four markers are tightly linked and are distributed within an approximate 82-kb region (Figure 1). D11S5028 is located ~19 kb upstream of the transcription start site, whereas the other three STRPs are located within gene introns.

We used TRANSMIT software to test for association in complete and incomplete families [13]. TRANSMIT is a generalization of the Transmission Disequilibrium Test (TDT) and uses the Expectation Maximization (EM) algorithm to estimate the missing information. The test statistic of TRANSMIT reduces to that of the TDT when families are full trios and markers are diallelic. When one parent and one affected sib (duos) or one affected and one healthy sib are available per family TRANSMIT can be used to perform the TDT analysis [14].

## Results and discussion

### Transmission disequilibrium test of markers

We evaluated the PITT and the NIMH series of pedigrees for association with the five polymorphic markers in the *ALG9 *gene. The TRANSMIT program provided the observed and the expected number of transmissions for each of the common alleles of the five markers from a parent to an affected offspring. The transmission patterns of the marker alleles to affected offspring did not show statistical significance. Specifically, in the PITT series, the SNP alleles encoding for Val289 and Ile289 were transmitted 185 and 85 times, similar to the expected transmission numbers of 187.5 and 82.5, respectively. Similarly, transmissions of the SNP alleles occurred 343 and 157 times in the NIMH series, which is very close to the expected numbers of 344 and 156, respectively. TRANSMIT provided χ^2^-based significance tests for transmission disequilibrium for each marker allele and for global (including all marker alleles) transmission disequilibrium. For robustness, the p-values associated with the global tests were computed via 1000 bootstrap samples. Bootstrap p-values were very close to the ones computed using the asymptotic χ^2 ^distribution of global tests. None of the common alleles of the five markers yielded statistically significant evidence for transmission disequilibrium. The results of the global χ^2 ^tests are presented in Table 2.

**Table 2:** Global TRANSMIT statistics for transmission disequilibrium tests of association between *ALG9 *variations and bipolar I

Pedigree Series	D11S5025 χ^2^_4 _(p-value)	V289I χ^2^_1 _(p-value)	D11S5026 χ^2^_4 _(p-value)	D11S5027 χ^2^_3 _(p-value)	D11S5028 χ^2^_2 _(p-value)
PITT	0.707 (0.93)	0.299 (0.58)	2.061 (0.57)	0.142 (0.98)	1.541 (0.46)
NIMH	0.385 (0.98)	0.012 (0.91)	2.17 (0.47)	0.349 (0.95)	0.396 (0.76)

### Transmission disequilibrium test of haplotypes

We wished to evaluate *ALG9 *further as a susceptibility gene for bipolar I using haplotype transmission disequilibrium tests. We tested D11S5028-V289I (rs10502151)-D11S5027 haplotypes in the PITT series and D11S5028-V289I (rs10502151) haplotypes in the NIMH series. These markers were selected for analyses because we were particularly interested in testing the haplotypic variations distinguishing the alleles encoding V289I (rs10502151), a common non-synonymous conservative substitution polymorphism. V289I (rs10502151) is biologically interesting because the amino acid Valine is conserved in the *ALG9 *gene in other species including fruit fly (*Drosophila melanogaster*), nematode (*Caenorhabditis elegans*), plant (*Arabidopsis thaliana*), fission yeast (*Schizosaccharomyces pombe*) and baker's yeast (*Saccharomyces cerevisiae*) [5]. We found non-random allelic associations among the markers (Table 3). The non-ancestral *ALG9 *allele encoding Ile289 was associated with the 110-bp and 372-bp alleles of D11S5028 and D11S5027, respectively (haplotypes 2.2 and 2.2.2 in Table 3). None of the haplotypes showed statistically significant evidence of transmission disequilibrium to offspring affected by bipolar I (Table 3). These results indicate that common allelic variations of the five markers than span *ALG9 *and haplotypic variations of V289I (rs10502151)-D11S5027 and D11S5028-V289I (rs10502151) are not associated with Bipolar I neither in the NIMH or the PITT pedigree series, which collectively contain 295 families showing transmissions to 385 affected offspring. These results are in agreement with our earlier results in the NIMH pedigree series obtained using broader diagnostic criteria and a different analytical approach [5].

**Table 3:** Allelic association of D11S5028-V289I-D11S5027 and haplotype transmission test

Pedigree series	Haplotype	D11S5028 Allele (bp)	V289I Allele GTA>ATA	D11S5027 Allele (bp)	Expected haplotype frequency (no allelic association)	Observed haplotype frequency*	Haplotype transmission disequlibrium p-value
PITT	1.1.1	106	G	366	0.13	0.27	0.78
	3.1.1	114	G	366	0.12	0.23	0.90
	4.1.1	116	G	366	0.03	0.07	0.70
	5.1.1	others	G	366	0.02	0.02	0.13
	2.1.4	114	G	Others	0.01	0.02	0.81
	2.2.2	110	A	372	0.02	0.22	0.82
	2.2.3	110	A	375	0.05	0.05	0.55
NIMH	1.1	106	G	-	0.19	0.29	0.67
	3.1	114	G	-	0.17	0.28	0.98
	4.1	116	G	-	0.04	0.05	0.62
	5.1	others	G	-	0.04	0.02	0.67
	2.2	110	A	-	0.11	0.30	0.78
	5.2	others	A	-	0.02	0.02	0.87

*Only observed-haplotypes that have frequencies more than 2% are shown because the uncommon haplotypes, that have frequencies less than 2%, are not analyzed by TRANSMIT.

To test whether the variations revealed by our multi-marker haplotypes represented the true haplotypic diversity in the *ALG9 *gene, we assessed its haplotype block structure in Caucasians in the HapMap database [15], because virtually all of our samples were of Caucasian origin. The common haplotypes of *ALG9 *in the HapMap database were defined by 18 intronic SNPs and the V289I (rs10502151) in 60 Caucasian samples (see Additional File 1). The non-ancestral allele encoding Ile289 (allele T of rs10502151) is associated with a common haplotype (HMB1) that has a frequency of 23%, whereas the remaining four haplotypes, HMA1, HMA1a, HMA2 and HMA2a, are associated with the ancestral allele encoding Val289 (allele C of rs10502151). Notably, only a single intronic SNP distinguishes HMA1 from HMA1a (SNP#19), and HMA2 from HMA2a (SNP#16), suggesting a recent common origin for these haplotype-duos. Thus, HMA1/HMA1a, HMA2/HMA2a and HMB1 define three common and distinct haplotype groups with frequencies of 45%, 28% and 23%, respectively. These three haplotype groups differ from each other in at least 7 SNP positions.

The *ALG9 *haplotype structure in the HapMap database is similar to the structure defined by D11S5028-V289I (rs10502151) or D11S5028-V289I (rs10502151)-D11S5027 in our dataset in that both approaches define three distinct and common haplotype groups (Table 3 and Additional File 1). Whereas the non-ancestral allele encoding Ile289 is associated with the common haplotypes 2.2.2 and 2.2, the ancestral allele encoding Val289 is associated with two common distinct haplotype groups 1.1.1 and 3.1.1 (three-marker haplotype) or 1.1 and 3.1 (two-marker haplotype) in our dataset (Table 3). The two common ancestral haplotype groups are tagged by the 106 bp and 114 bp-alleles of D11S5028, whereas the non-ancestral haplotypes are tagged by the 110 bp-allele (Table 3), indicating that the repeat alleles of the three haplotypes differ by at least 4 bp (2 units). Because (CA)n repeat mutations cause an increase or decrease in repeat number by one unit [16], we predict that the two ancestral and one non-ancestral haplotype groups are phylogenetically distant and correspond to the same three haplotype groups defined in the HapMap database.

Whereas the two ancestral haplotypes in the HapMap database, HMA1/HMA1a and HMA2/HMA2a, have frequencies of 45% and 28% respectively (Additional File 1), those in our study have frequencies of 27% and 23% (three-marker haplotypes) or 29% and 28% (two-marker haplotypes) (Table 3). The non-ancestral haplotypes in the HapMap database (HMB1) and in our study (2.2.2 or 2.2) have comparable frequencies of 23% and 22 or 30%, respectively. Thus, the frequencies of the common haplotypes defined by D11S5028-V289I (rs10502151)-D11S5027 or D11S5028-V289I (rs10502151) and HapMap are comparable. Nevertheless, it is conceivable that if there is a susceptibility-variant in the most common ancestral haplotype in HapMap database (HMA1/HMA1a), our method would have reduced power to detect it because the frequency of the most common ancestral haplotypes (1.1.1 or 1.1) in our dataset is lower than that in the HapMap database (27% or 29% vs. 45%), thus reducing the number of informative chromosomes tested for association with bipolar I disorder. If, however, there is a rare susceptibility variant on haplotype 1.1.1 or 1.1, our approach would have more power to detect it because of the stronger allelic association of the susceptibility variant with haplotype 1.1.1 or 1.1 than with HMA1/HMA1a.

If the *ALG9 *disruption is causally linked to the development of BPAD in the translocation family, several other reasons could be invoked to explain our present failure to detect an association between *ALG9 *and BPAD. It is conceivable that rare loss-of-function mutations and gross gene aberrations of *ALG9 *that have dramatic functional consequences, rather than the common allelic and haplotypic variations, could increase predisposition to BPAD. Here, we tested two series of pedigrees. The NIMH pedigrees are enriched for families with multiple affected individuals, whereas 129 of 135 (95.6%) of the PITT pedigrees contain a single affected individual. If rare susceptibility variants in *ALG9 *contribute to the familial enrichment of BPAD in the NIMH pedigrees, association testing by common alleles and haplotypes would have low power to detect it. Although the PITT series is primarily consisted of small nuclear families with a single affected individual, and therefore might be more suited to detect common, low-penetrance susceptibility variants, its sample size may not be large enough if the relative risk of susceptibility conferred by the common variant is low. It is also conceivable that certain environmental factors or sex-specific influences may modify the risk conferred by an *ALG9 *variant. Similar mechanisms may have played a role in failure to support an association between *DISC1 *variations and schizophrenia in some studies [17-20], although an increasing amount of evidence supports a role for *DISC1*, a gene disrupted by a translocation breakpoint in an extended family, in the etiology of schizophrenia.

## Conclusion

In conclusion, our further analysis of *ALG9 *gene variations failed to support a major role in predisposition to BPAD. Nevertheless, we cannot rule out a role for *ALG9 *in BPAD susceptibility because of the limitations of the markers and the pedigrees analyzed here. Interestingly, another protein N-glycosylation pathway gene, *MGAT5 *(Mannoside acetylglucosaminyltransferase 5) at chromosome band 22q21, was also found to be disrupted by an unbalanced translocation breakpoint in a schizophrenia case [2]. Thus, the structural aberrations of sugar transferase genes in BPAD and schizophrenia and the recent findings that the germ line homozygous mutations of *ALG9 *leads to progressive microcephaly, cerebral and cerebellar atrophy underscores the significance of N-glycosylation pathway in central nervous system homeostasis and may warrant further analyses of *ALG9 *and other mannosyltransferase genes in the etiopathogenesis of psychosis.

## Abbreviations

BPAD (Bipolar Affective Disorder), CDG (Congenital Disorders of Glycosylation), *DIBD1 *(Disrupted in Bipolar Disorder), Ile/I (Isoleucine), IRB (Institutional Review Board), LLO (lipid-linked oligosaccharides), NIMH (National Institute of Mental Health), PITT (University of Pittsburgh), SNP (Single Nucleotide Polymorphism), STRP (simple tandem repeat polymorphism), TDT (Transmission Disequilibrium Test), Val/V (Valine).

## Competing interests

The author(s) declare that they have no competing interests.

## Contributors

BEB designed the study, obtained funding and drafted the manuscript, JEWB performed genotyping experiments and data entry, SAB performed statistical analyses, SDW and VLN provided pedigree information and the DNAs. All authors contributed to the manuscript and approved its final version.

## Supplementary Material

Additional File 1
